# Surface modification of nanoparticles enables selective evasion of phagocytic clearance by distinct macrophage phenotypes

**DOI:** 10.1038/srep26269

**Published:** 2016-05-19

**Authors:** Yaqing Qie, Hengfeng Yuan, Christina A. von Roemeling, Yuanxin Chen, Xiujie Liu, Kevin D. Shih, Joshua A. Knight, Han W. Tun, Robert E. Wharen, Wen Jiang, Betty Y.S. Kim

**Affiliations:** 1 Department of Neurosurgery, Mayo Clinic College of Medicine, 4500 San Pablo Road, Jacksonville FL, 32224, USA; 2 Department of Orthopedics, Zhongshan Hospital, Fudan University, 111 Yixueyuan Road, Xuhui, Shanghai, China; 3 Mayo Graduate School, Mayo Clinic College of Medicine, 200 1st Street SW, Rochester, MN, 55902, USA; 4 Department of Hematology/Oncology, Mayo Clinic College of Medicine, 4500 San Pablo Road, Jacksonville FL, 32224, USA; 5 Department of Cancer Biology, Mayo Clinic College of Medicine, 4500 San Pablo Road, Jacksonville FL, 32224, USA; 6 Department of Radiation Oncology, MD Anderson Cancer Center UT-MD Anderson Cancer Center, 1515 Holcombe Blvd Houston TX, 77030, USA; 7 Department of Neuroscience, Mayo Clinic College of Medicine, Mayo Clinic College of Medicine, 4500 San Pablo Road, Jacksonville FL, 32224, USA.

## Abstract

Nanomedicine is a burgeoning industry but an understanding of the interaction of nanomaterials with the immune system is critical for clinical translation. Macrophages play a fundamental role in the immune system by engulfing foreign particulates such as nanoparticles. When activated, macrophages form distinct phenotypic populations with unique immune functions, however the mechanism by which these polarized macrophages react to nanoparticles is unclear. Furthermore, strategies to selectively evade activated macrophage subpopulations are lacking. Here we demonstrate that stimulated macrophages possess higher phagocytic activities and that classically activated (M1) macrophages exhibit greater phagocytic capacity than alternatively activated (M2) macrophages. We show that modification of nanoparticles with polyethylene-glycol results in decreased clearance by all macrophage phenotypes, but importantly, coating nanoparticles with CD47 preferentially lowers phagocytic activity by the M1 phenotype. These results suggest that bio-inspired nanoparticle surface design may enable evasion of specific components of the immune system and provide a rational approach for developing immune tolerant nanomedicines.

The body’s innate immune system plays a critical role in mediating the host’s defense against foreign pathogens^1^. Macrophages are derived from the monocytic lineage precursor cells that are important for both the innate and adaptive immune responses. As the main scavenger cells of the body, macrophages possess the unique ability to engulf foreign particulates, cellular debris and stressed cells in order to maintain cellular homeostasis as well as immune surveillance within the innate immune system. Macrophages are also important linkers for adaptive immunity *via* antigen processing, presentation and subsequently T lymphocyte priming^2^. Their significance within the immune system is further exemplified by their heterogeneity and plasticity, with many subsets of macrophage populations exhibiting specialized and polarized functional capabilities of regulating tissue inflammation and phagocytic clearance^3^. In their simplest form, polarized macrophages are divided into classically activated M1 cells and alternatively activated M2 cells, dependent on their exposure to specific microbial stimuli such as lipopolysaccharide (LPS) or cytokines such as interleukin-4 (IL-4), interleukin-10 (IL-10) or interferon-γ (IFNγ)^4^. Functionally, these macrophage phenotypes are distinct in their membrane expression levels of important phagocytosis receptors such as the opsonic receptor CD16 and mannose receptor; in their cytokine and chemokine production; and in their ability to facilitate or suppress inflammation, scavenge debris and promote tissue repair^5^. Given their integral role within the immune cascade, a complete understanding of how nanomaterials interact with the monocyte-macrophage system and, in particular, with distinct polarized macrophage phenotypes, is crucial to the clinical translation of nanomedicine. More importantly, the ability to design nanomaterials that can selectively target or evade specific macrophage phenotypes would bring us one step closer toward the development of tailored nanomedicine platforms that are safe and immune tolerant.

In the current study, we examined the phagocytic capacities of polarized M1 and M2 macrophages to different sized nanoparticles and surface modifications. We hypothesized that these uniquely polarized macrophage populations possess differential capabilities to engulf nanoparticles compared to their non-activated M0 counterpart as well as to each other. We then studied the effects of surface coating chemistry using conventional techniques such as polyethylene glycol (PEG) on the phagocytic clearance of nanoparticles. Finally, we modified the nanoparticle surface with specific biomolecules and demonstrated, for the first time, that alteration of the phagocytic signalling cascade can selectively inhibit nanoparticle phagocytosis by uniquely polarized macrophage subsets.

## Results

### Nanoparticle modification and characterization

**Figure 1:**
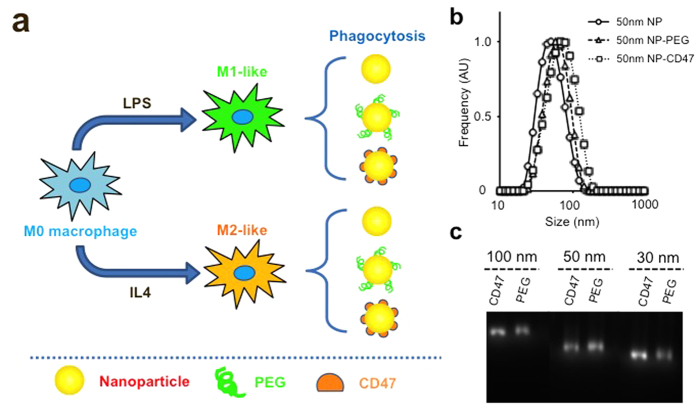
Experimental setup and nanoparticle characterizations. (**a**) Schematic showing polarization of macrophages using different stimuli. The polarized macrophages are then incubated with nanoparticles modified with unique surface chemistry to assess for their phagocytic activities. (**b**) Hydrodynamic diameter measurements of 50 nm nanoparticles with different surface modifications. (**c**) Gel electrophoresis assay of different sized nanoparticles after surface modification with PEG or recombinant CD47 protein. The narrow bands from the gel image demonstrate the monodispersity of the final conjugates.

To study the phagocytic efficiency of polarized macrophage subpopulations to various sized nanoparticles, we used carboxylic acid terminated fluorescently labeled polystyrene nanoparticles as a model system. We selected nanoparticles of three different sizes with hydrodynamic diameters of 30 nm, 50 nm and 100 nm. These nanoparticles were then conjugated with either 10 K molecular weight amino-PEG or mouse recombinant CD47 and incubated with specific polarized macrophage populations (Fig. 1a). Unmodified and surface-modified nanoparticles (amino-PEG or CD47 conjugated nanoparticles) were highly monodisperse (Fig. 1b,c) with similar negative surface charge profiles (Supplementary Table 1). As expected, the modification of nanoparticles with amino-PEG and CD47 slightly increased the final hydrodynamic diameter (Fig. 1b, Supplementary Table 1). The fluorescence intensities of the nanoparticles were stable following surface modification and with exposure to different mediums, thus enabling us to quantitatively measure particle uptake for later experiments (Supplementary Figure 1).

### Polarization of macrophages affects nanoparticle phagocytosis

**Figure 2:**
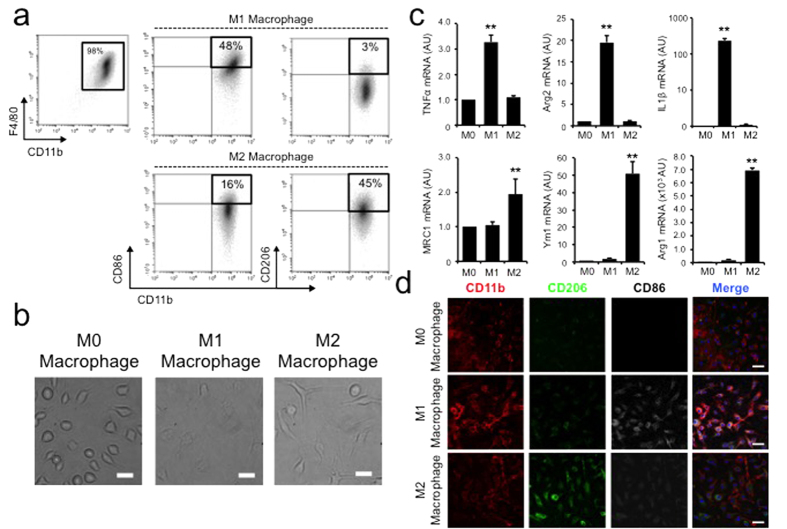
Characterization of macrophage polarization. (**a**) Flow cytometry analysis of polarized macrophage population using phenotypic surface markers such as CD86 (M1) and CD206 (M2). (**b**) The polarized macrophages possess unique physical appearance with M1 macrophages exhibit more oval while M2 macrophage demonstrate elongated spindle-like morphologies. Scale bar = 20 μm (**c**) Macrophage polarization is further confirmed by their cytokine productions. LPS stimulated macrophage showed marked higher expression of M1 cytokines (TNFα, Arg2 and IL1β) while IL4 stimulated macrophages had higher expression of M2 cytokines (MRC1, Ym1 and Arg1). ** denotes p < 0.05, n = 5. (**d**) Confocal microscopy further demonstrates differential surface expression of M1 and M2 phenotypic markers. Scale bar = 20 μm.

Next, we tested whether differentially polarized macrophages possess distinctive capacities to take up nanoparticles of different sizes. Circulating monocytes are activated by different stimuli into macrophage phenotypes that specialize in unique immune functions. Classically activated M1 macrophages are often believed to be microbicidal and pro-inflammatory while alternatively activated M2 macrophages predominantly function as immune modulators^4^. To study how these different macrophage populations respond to nanoparticles, we isolated fresh bone marrow-derived cells from 6–8 weeks old C57BL/6J immune-competent mice using a well established protocol^6^. The isolated bone marrow cells were cultured for 7 days in bone marrow-derived macrophage (BMM) complete medium to remove contaminant non-adherent cells of other lineages. Adherent bone marrow-derived macrophages had a purity greater than 98% based on flow cytometry analysis using classic macrophage markers F4/80 and CD11b^7^,^8^. These bone marrow derived macrophages (M0) were then activated into M1 or M2 phenotypes with LPS +/− IFNγ or IL4 (Fig. 1a)^9^. The M1 and M2 macrophages exhibited preferential expression of classic phenotypic surface markers such as CD86 and CD206, respectively (Fig. 2a,d). Morphologically the two subtypes were different: M1 macrophages were oval in shape while M2 macrophages were elongated with spindle-like morphology consistent with previous reports (Fig. 2b)^10^. Given that a key measure of macrophage activation is the production of different inflammatory cytokines, we also demonstrated that M1 activated macrophages had significantly elevated production of TNFα, Arg2 and IL1β^11^,^12^, whereas M2 activated macrophages demonstrated increased production of MRC1, Ym1 and the prototypical alternative activation marker Arg1 (Fig. 2c).

**Figure 3:**
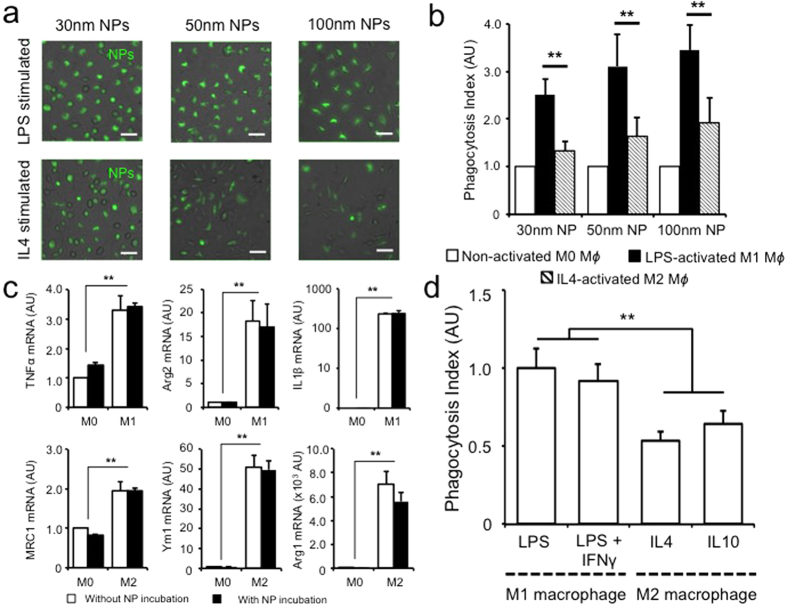
Preferential uptake of nanoparticles by M1 polarized macrophages. LPS stimulated M1 macrophages demonstrate increased nanoparticle uptake across all tested sizes as demonstrated by microscopy (**a**) and flow cytometry (**b**). Phagocytosis index is defined as the mean fluorescence intensity per cell. Uptake of nanoparticles by activated macrophages did not change their polarization status based on their cytokine expression profile (**c**). Macrophage polarization with other Th1 or Th2 cytokines showed similar predominant nanoparticle uptake among M1 cells (**d**). ** denotes p < 0.05. Scale bar −50 μm, n = 3.

M1 macrophages activated by IFN-γ and/or LPS stimulation have been reported to experience enhanced phagocytosis of bacterial pathogens via increased complement secretion and up-regulation of surface complement receptors^13^. In contrast, IL4 stimulated M2 macrophages were reported to have increased capacity for fluid-phase pinocytosis and MRC1 dependent or independent endocytosis^14^. How these phenotypic and functional differences between M1 and M2 macrophages affect nanoparticle clearance is unclear. When incubating different sized nanoparticles with macrophages, M1 and M2 cells showed increased uptake capacities compared to their non-stimulated M0 counterparts across all sizes (Fig. 3a,b, Supplementary Figure 2). Interestingly, the degree of nanoparticle internalization by M1 macrophages was significantly higher than by M2 macrophages (Fig. 3b, Supplementary Figure 2). Combined with similarly observations with uncoated gold nanoparticles and latex beads, these findings further support the differential nanoparticle uptake by M1 and M2 cells mediated through a complex process involving cytoskeletal remodeling, membrane fusion and vesicular transport^15^,^16^,^17^. To confirm that the observed nanoparticle uptake is indeed mediated via energy dependent internalization rather than non-specific absorption to the cellular membrane, incubation studies of nanoparticles at 4 °C and in the presence of NaN_3_ pretreatment were performed (Supplementary Figure 3). A significant decrease in nanoparticle uptake capacity was observed, suggesting that phagocytic process is the predominant process contributing to the observation of nanoparticle uptake. Although the chronicity of macrophage stimulation may affect its phagocytic ability, it has been suggested that 24 hours of stimulation is enough to activate the macrophages in the classical pathway^9^. Consistent with this observation, we noted that nanoparticle uptake by both M1 or M2 macrophages showed no significant difference despite prolonged stimulation beyond 24 hours (Supplementary Figure 4).

### Phagocytosis of nanoparticle does not affect macrophage polarization

Macrophages harbor significant plasticity and heterogeneity. Activated macrophages can easily polarize from one phenotype to another in a reversible manner based on environmental stimuli^4^,^18^. To assess whether nanoparticles possess inflammation modulatory potential, we examined the gene expression profiles of classic markers in M1 and M2 macrophages, and demonstrated that their expression levels were not altered after nanoparticle incubation (Fig. 3c).

Given the complexities of macrophage activation and the unique functional differences between polarized macrophage phenotypes, further sub-classifications of M1 and M2 macrophages have been proposed^19^. In the case of classically activated M1 macrophages, M1a macrophages stimulated by IFNγ together with LPS were shown to exhibit enhanced phagocytic and bactericidal capacities compared to innate or M1b macrophages stimulated via LPS or other pathogen-associated molecular patterns alone despite their phenotypic similarities^20^. Similarly, alternatively activated M2 macrophages can be further divided into M2a, M2b and M2c subtypes, where M2b and M2c cells function as regulatory macrophages with elevated production of immune suppressive cytokine IL10^21^. To examine whether nanoparticle uptake is different among these macrophage subpopulations, we stimulated M0 macrophages with different combinations of cytokines and bacterial proteins (Fig. 3d). M1 macrophages maintained their superior nanoparticle phagocytosis capacity over M2 macrophages, and we did not observe significant difference among sub-populations of classically or alternatively activated macrophages (Fig. 3d).

### PEGylation reduces nanoparticle uptake by all macrophage populations

**Figure 4:**
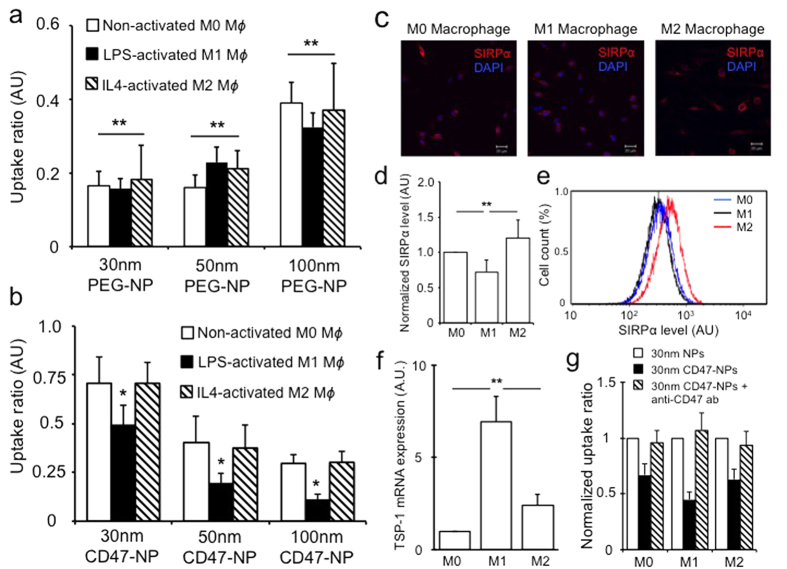
Surface modification of nanoparticle affects their uptake by macrophages. PEGylation of nanoparticles reduced their uptake by all macrophage populations (**a**). Nanoparticle surface modification with mouse recombinant CD47 protein preferentially reduced particle uptake within M1 population (**b**). Uptake ratio defined as the ratio median nanoparticle uptake per cell for surface modified nanoparticles versus uncoated nanoparticles. M1 macrophages stimulated by LPS demonstrated decreased SIRPα expression by immunofluorescence, qPCR and flowcytometry (**c–e**). M1 cells however, showed higher TSP-1 expression level than M0 and M2 macrophages (**f**). Blocking of CD47-SIRPα interaction via CD47 antibodies abrogated the phagocytosis inhibitory effect (**g**). Uptake ratio is defined as the ratio of mean fluorescence intensity per cell of surface modified nanoparticle to non-modified nanoparticles. * & ** denote p < 0.05, scale bar = 20 μm, n = 3.

Nanoparticle surface modification with long-chain polymers such as PEG has been shown to decrease nonspecific serum protein adsorption onto the nanoparticle surface, decrease their phagocytic uptake and prolong circulation time of synthetic nanomaterials^15^,^22^. To examine how PEGylation of nanoparticles affects their phagocytic clearance by different macrophage populations, we incubated inactivated M0 and activated M1 and M2 macrophages with nanoparticles coated with 10,000 molecular weight PEG. Interestingly, PEGylation reduced nanoparticle uptake by all macrophages, irrespective of their polarization status (Fig. 4a, Supplementary Figure 5a,b). The fact that PEG coating reduces the adsorption of a wide range of soluble proteins such as complements, glycosylated proteins, and lipoproteins may have contributed to the generic uptake reduction across all macrophage populations^15^,^23^. We also observed that PEGylation of our polystyrene based-nanoparticles results in a similar size-dependent uptake profile by macrophages as previously demonstrated with PEG-grafted gold nanoparticles (Fig. 4a)^15^.

### Surface modification with CD47 preferentially reduces M1 macrophage phagocytosis

Phagocytosis of cellular debris and particulates is governed by a series of complex molecular mechanisms initiated by the recognition of pro-phagocytosis signalling ligands by their receptors. The integrin-associated transmembrane protein CD47 was previously shown to be up-regulated in cells of hematopoietic lineage as well as multiple cancer cells to evade phagocytic clearance by resident macrophages^24^,^25^. CD47 interacts with signal regulatory protein α (SIRP α) expressed by macrophages and dendritic cells^26^. The binding of SIRPα with CD47 results in the phosphorylation of the cytoplasmic tail of SIRPα leading to the binding and activation of protein phosphatases that block phagocytosis, possibly through the inhibition of motor protein myosin-IIA accumulation at the phagocytic synapses^25^,^27^. Nanoparticles greater than 100 nm coated with synthetic peptides designed from CD47 protein were noted to have prolonged circulation time *in vivo*^28^. To investigate the effects of CD47 coating on nanoparticle uptake by different macrophage populations, we incubated non-stimulated M0 macrophages and stimulated M1 and M2 macrophages with nanoparticles conjugated with mouse recombinant CD47 protein. Similar to PEGylated samples, nanoparticle surface modification with CD47 significantly reduced phagocytosis across all macrophage populations (Fig. 4b, Supplementary Figure 5c). Interestingly, a markedly reduced uptake of CD47 coated nanoparticles was observed in M1 compared to M0 or M2 macrophages (Fig. 4b, Supplementary Figure 5c).

### CD47-SIRPα interaction suppresses nanoparticle phagocytosis

Given the important interaction of CD47-SIRPα, we set out to determine whether the observed differential phagocytosis of CD47-nanoparticles is mediated by SIRPα signalling. Interestingly, immunofluorescence imaging, flow cytometry analysis and PCR all showed that classically activated M1 macrophages had decreased expression of SIRPα protein on their surface compared to both M0 or alternatively activated M2 macrophages (Fig. 4c–e, Supplementary Figure 6). This appeared to be consistent with previous studies involving LPS stimulated macrophages^29^,^30^, as SIRPα is primarily an immune suppressive protein that inhibits innate immune response activation and inflammation^31^. Given that CD47 is also known to bind thrombospondin-1 (TSP-1), an extracellular matrix protein, we tested whether its expression level differed within activated M1 or M2 macrophages. Although activated macrophages have been demonstrated to have higher TSP-1 expression compared to M0 cells, we found that TSP-1 production was much higher in M1 macrophages than M2 cells (Fig. 4f, Supplementary Figure 7), consistent with prior observation showing elevated TSP-1 expression via stimulation by bacterial proteins and is associated with pro-inflammatory effects^32^,^33^. Furthermore, TSP-1 has previously been proposed to complement SIRPα and CD47 interaction in a coordinated fashion to regulate phagocytosis of aged and dying hematopoietic cells^34^.

To confirm that our observed nanoparticle uptake reduction by activated macrophages is indeed mediated through CD47-SIRPα interaction, we incubated CD47 coated nanoparticles with different macrophages in the presence of CD47 blocking antibody (Fig. 4g). Treatment with CD47 blocking antibodies reduced the amount of phagocytosis inhibition observed with CD47-coated nanoparticles, suggesting a necessary role of CD47 in promoting nanoparticle uptake evasion (Fig. 4g). Interestingly, when macrophages are treated with uncoated nanoparticles and free recombinant CD47, we also noted a similar pattern of decreased nanoparticle uptake but to a lesser degree than CD47-coated nanoparticles (Supplementary Figure 8), which raises the possibility that multivalent binding may be contributing to a stronger stimulation of SIRPα receptor signalling and the downstream biological response, as we have demonstrated previously with ligand coated nanoparticles targeting cell membrane receptors^35^.

## Discussion

Macrophages are professional phagocytic cells within the body that play an indispensable role in the immune system with decisive functions in both innate and acquired immunity. Although previous studies have looked into the interactions between macrophages and nanoparticles both *in vitro* and *in vivo*, studies on nanoparticle uptake by heterogeneous macrophage subpopulations are limited^15^,^28^,^36^,^37^. A recent study showed that PEGylated cylindrical nanoparticles greater than 300 nm appeared to have different pharmacokinetic profiles in Th1 or Th2 dominant mouse strains, suggesting that the polarization of MPS by Th1 or Th2 cytokines can influence nanoparticle clearance^38^. Subsequently, others investigated uptake capabilities of polarized macrophages using immortalized cell lines from tumor extracts with silica or chemically modified Cowpea mosaic virus particles^39^,^40^. In these studies, it was observed that alternatively activated THP1 cells showed enhanced phagocytosis activity compared to classically activated cells. However, the applicability of these observations to primary bone marrow derived macrophages is unclear, given previous reports demonstrating that bone marrow derived macrophages appeared to exhibit opposite response when exposed to latex nanoparticles after IL4 stimulation via subsequent STAT6 signalling^16^.

In this study we observed enhanced nanoparticle uptake by activated macrophages. More importantly, the polarization of macrophages towards the M1 phenotype resulted in enhanced nanoparticle uptake for all sizes. Using a mouse primary bone marrow culture, we studied sub-100 nm nanoparticle uptake by polarized macrophages using an established protocol. In this setting, we observed that classically activated M1 macrophages appeared to exhibit an increased uptake ability of non-PEGylated nanoparticles compared to their M2 counterparts. Consistent with previous studies, nanoparticles coated with PEG resulted in reduced phagocytosis across all macrophage phenotypes and nanoparticle sizes. However, smaller nanoparticles experienced greater internalization inhibition compared to larger nanoparticles, in keeping with previous reports^15^. In contrast, when we coated our nanoparticles with mouse recombinant CD47 (‘don’t eat me protein’), we observed a greater reduction of large nanoparticle uptake as well as a larger inhibition of uptake by M1 macrophages, suggesting that CD47 signalling appears to play a greater role in regulating phagocytosis in M1 compared to M2 cells. This hypothesis is further supported by a recent study demonstrating inhibition of CD47-SIRPα interaction by anti-CD47 antibodies produced a higher pro-phagocytosis rate of cancer cells by M1 as compared to M2 macrophages^41^. A significant amount of studies have been performed looking at the role of surface coating on biological interactions of nanoparticles^42^,^43^. Extensive studies have also examined the role of surface chemistry on serum protein absorption onto nanoparticles and its role in promoting cellular uptake^44^,^45^. All of these are strongly dependent upon the local environment surrounding the nanoparticles which is dictated by both culture conditions and production of secreted proteins by cultured cells. Our study adds to the current understanding that in addition to physical and chemical properties of the nanoparticle and the local environment, specific cell populations with distinctive phenotypic functions may also play a critical role in regulating nanoparticle uptake.

Phagocytosis is a complex process that requires coordinated actions of multiple receptors, ligands and intracellular signals. Although the blockade of CD47-SIRPα interaction alone is not sufficient to induce phagocytosis in the absence of additional pro-phagocytic signals such as binding of ligands with Fc, complement or scavenger receptors^46^, CD47-SIRPα interaction in itself may be sufficient to inhibit phagocytosis. However, a complete inhibition of phagocytosis does not necessarily equate to eliminating nanoparticle uptake by phagocytes, as additional endocytic and pinocytic processes can also promote particle engulfment^14^. While fluid phase uptake such as micropinocytosis or pinocytosis, and to a certain extent receptor-mediated endocytosis, results in the uptake of relatively small particulates and cellular debris, phagocytosis plays a more prominent role in the clearance of larger particles. Therefore, it is not surprising that we observed greater anti-phagocytic benefits of CD47 coating as the nanoparticle sizes increased. At the same time, PEGylation appeared to favour evasion of nanoparticle uptake in the smaller size range^15^. Although CD47-mediated anti-phagocytosis is thought to arise from inhibition of myosin II accumulation and inactivation of actomyosin contraction, which results in targets being ‘pulled into’ macrophages, its effect on sub-100 nm nanoparticles is surprising and was speculated to involve additional physical parameters such as shape and particle flexibility^47^. Consequently, these results raise the interesting prospect that surface chemistry design requires individualized tailoring to take the nanoparticle’s physical parameters such as size into consideration in order to yield optimal evasion of MPS clearance. For intermediate sized nanoparticles between 10–100 nm, both endocytic and phagocytic processes may contribute to their clearance, thus supporting a combinatorial coating strategy to minimize both processes on nanoparticle uptake.

Finally, we observed that CD47-coating preferentially decreased nanoparticle uptake by M1 macrophages, despite a lower expression of SIRPα on these cells. CD47, as a member of the integrin associated protein family, can bind to multiple cell surface and extracellular proteins and receptors. Previous studies have demonstrated that CD47, in fact, acts as a phagocytosis switch, dependent on its precise interactions with SIRPα and TSP-1^48^. While CD47- SIRPα interaction is the predominant phagocytosis inhibitory signal for macrophages, TSP-1 also plays a important role in regulating the phagocytosis axis. This is especially the case during pre-apoptotic plasma membrane re-organization, which results in alteration of CD47 distribution^48^. Therefore, the net anti-phagocytosis response observed in our CD47 coated nanoparticles by M1 macrophages is likely, at least in part, due to complex interactions and signalling by CD47, SIRPα, and TSP-1, which are differentially expressed and regulated within each macrophage subpopulation. Furthermore, additional scavenger receptors and yet to be discovered potential interactions involving other integrin binding proteins may also contribute to this process.

Distinctively activated macrophage populations possess unique pro- and anti-inflammatory properties that play important roles in immune regulation and disease pathology. During strokes, for example, classically activated M1 macrophages were shown to promote inflammation after ischemic injury and release cytotoxic cytokines that lead to increased neuronal death^49^. In contrast, M2-like tumor associated macrophages promote immune suppression and facilitate tumor evasion of host immune surveillance^50^. Therefore, the understanding of how nanomaterials interact with specific macrophage phenotypes and the ability to design nanomaterials that can selectively target or evade these macrophage subpopulations is vital to the field of nanomedicine research. One thing to note is that macrophage polarization represents a continuum of different phenotypic states and M1/M2 paradigm is the simplest model to represent such^4^. Other intermediate states of macrophage polarization may exhibit distinctive behaviors that deviate from the model used here. Our current study demonstrates that activated primary bone marrow macrophages have increased phagocytic capacity for nanoparticles. In particular, classically activated M1 macrophages exhibit greater nanoparticle uptake potential compared to alternatively activated M2 macrophages. Surface modification strategies using PEGylation can reduce nanoparticle uptake by these cells. However, CD47 modified nanoparticles showed a prominent anti-phagocytosis property in M1 cells that may in part be due to the complex signalling interactions of CD47 with SIRPα and TSP-1. Although our observation in bone marrow derived macrophages is interesting, whether similar conclusion can be applied to monocyte derived tissue specific macrophages remains to be seen. For example, how the M1/M2 polarization paradigm applies to tissue-specific macrophages such as microglia in the central nervous system and its role in mediate neuroinflammation remains under investigation^51^. Similarly, resident macrophages within the liver (Kupfer cells), spleen, or lung (alveolar macrophages) may also possess unique ability to clear nanoparticles that is different from their bone marrow derived counterpart. A complete understanding of the molecular interactions that regulate nanoparticle uptake by professional phagocytes would provide significant opportunities to develop immunologically smart nanomaterials that can take full advantage of the diversity of the mononuclear phagocyte system.

## Methods

### Animals

All C57BL/6J mice are maintained at the animal facility of Mayo Clinic in Florida in specific-pathogen-free environment. Typically 6 to 8 weeks mice were used for the study. All experiments were conducted in accordance with the guidelines of institutional IACUC and were approved by Mayo Clinic Florida animal care committee.

### Regents

All chemicals used for conjugations including N-Hydroxysulfosuccinimide (sulfo-NHS), N-(3-Dimethylaminopropyl)-N′-ethylcarbodiimide (EDC), and amino-polyethylene glycol 10 K (amino-PEG) were purchased from Sigma-Aldrich (St. Louis, MO). All cell culture reagents, including Dulbecco’s Modification of Eagle’s Medium (DMEM) (Corning), L929 medium (Stony Brook Cell Culture/Hybridoma facility), Sodium pyruvate (Gibco), Penicillin-Streptomycin (PS) Solution (Gibco), Lipopolysaccharides from *Escherichia coli* (Sigma), recombinant mouse IL4 (Sigma), PureLink RNA Mini Kit (ABI), High-Capacity cDNA Reverse Transcription Kit (ABI), Taqman gene expression assay kit (ABI) were purchased separately from vendors. For immunostaining, anti-mouse FITC-CD206, anti-mouse PE-CD86, anti-mouse PE-cy7-F4/80, and anti-mouse APC-CD11b were purchased from Biolegend (San Diego, CA). Mouse recombinant CD47 protein was purchased from R&D systems (Minneapolis, MN), mouse anti-CD47 monoclonal antibodies were purchased from Affymetrix (clone MIAP 301) and generated using hybridoma established previously (clone MIAP 410)^52^. Mouse SIRPα staining and neutralizing antibodies were purchased from Biolegend and Novusbio, respectively.

### Nanoparticle Characterization

Fluorescently labeled carboxylated polystyrene nanoparticles of different sizes (30 nm, 50 nm, 100 nm) were purchased from Magsphere (Pasadena, CA). These nanoparticles have an excitation peak of 538 nm and an emission peak at 584 nm. Solution-based characterizations of nanoparticle size and charge distributions were measured using Zeta-Sizer Nano instrument (Malvern, MA). Gel electrophoresis of nanoparticles after conjugation were performed with 0.5–1% Agarose gel, running at 100 V for 45 min and imaged with gel imager (Typhoon, GE healthcare, US). Fluorescence intensity measurements of the nanoparticles were performed using the Nanodrop Fluorospectrometer (Thermo Scientific).

### Nanoparticle Conjugation

Conjugation of nanoparticles with recombinant CD47 protein and amino-PEG were done according to previously established protocols using carbodiimide-mediated chemistry^53^. Briefly, different sized nanoparticles are diluted in PBS to a final concentration of 100 nM (based on the stock solution of 2.5% solid). A 1:10 ratio of nanoparticle: amino-PEG/CD47 molar mixture was reacted with a 100:1 molar ratio of EDC/sulfo-NHS:nanoparticle for 2 h. The resulting solution was then washed 2 times with 50 K molecular weight cut-off (MWCO) centrifugal filter (Millipore, US) at 3000 RPM for 5 min to remove excess unreacted molecules. The resulting pellet is then centrifuged down at 10000 RPM for 10 min and re-suspended in PBS. Concentrations of CD47 and PEG after conjugation are measured using Bradford or iodine assay.

### Bone Marrow Macrophage Isolation

Bone marrow macrophage extraction was performed according to previously established protocol^6^. Briefly, C57BL/6J mice were sacrificed by CO_2_ asphyxiation followed by cervical dislocation. The femurs and tibias were then surgical removed after striping away skeletal muscles by forceps, and the bone marrow was flushed with PBS using a 10 ml syringe and a 25-gauge needle. The bone marrow cells were mixed into a single-cell suspension using a pipet. The cells were then washed with PBS and re-suspend in bone marrow derived macrophage (BMM) complete medium (50 ml solution consists of: 25 mL DMEM, +15 mL L929 + 7.7 mL FBS + 0.5 mL sodium pyruvate + 1.8 mL PS). The bone marrow cells are counted and plated at a concentration of 2–3 × 10^6^ cells per 10 cm plate in the BMM complete medium. The cells are then incubated at 37 °C with 5% CO_2_. Fresh culture medium was changed at day 3 and day 5. Cells are harvested on day 7 for subsequent experiments.

### Macrophage Activation

Bone marrow derived monocytic cells were activated according to previously published protocol^9^. For classically activated M1 macrophages, bone marrow cells were incubated with fresh BMM complete medium,with 100 ng/ml LPS or 100 ng/ml LPS and 25 ng/ml IFNγ for 24 or 48 hrs. For M2 polarization, cells were cultured with BMM complete medium supplemented with 10 ng/ml of IL4 (M2a) or 10 ng/ml of IL10 (M2c) for 24 or 48 hrs.

### RNA Extraction and Analysis

Total cellular RNA was extracted from macrophage using commercially available RNA kit according to the manufacturer’s instruction (ABI). RNA quality was assessed using the Nanodrop spectrophotometer (Thermo scientific). For quantitative PCR, cDNA was synthesized from total RNA using the high-capacity cDNA reverse transcription kit (ABI). Gene expression was measured by the change-in-threshold (ΔΔCT) method based on quantitative PCR using the ABI 7900HT real-time system (Thermo Fisher Scientific, US). TagMan primer sets for TNFα, Arg1, Arg2, YM1, IL1β and Mrc1, SIRPα, TSP-1 were used.

### Flow Cytometry

Cells of interest were harvested and stained for 30 min with gentle shaking at 4 °C with primary antibodies targeting plasma surface markers such as CD11b, CD11c, F4/80 and CD86. The cells are then washed and fixed with fixation buffer containing 1X PBS with 4% paraformaldehyde (BioLegend, San Diego), and resuspended in permeabilization buffer (Biolegend, San Diego). The cells then underwent staining for intracellular marker (CD206) for 15–20 min at room temperature. For SIRPα expression analysis, macrophages are stain with percp-cy5.5 SIRPα (BioLegend, San Diego) according to manufacturer’s recommendations. Non-phagocytic murine tumor cell line N_2_O_2_ and TUBO are used as negative controls. All flow cytometry analyses were performed on the FACS Aria flow cytometer (BD Bioscience) and data were analyzed by Flowjo (TreeStar) and BD Diva software.

### Phagocytosis Assay

Phagocytosis assays of different macrophage sub-populations were performed by incubating different sized nanoparticles with macrophages at a concentration that is consistent with their normalized total surface area per cell. The concentrations of the nanoparticles were determined by fitting to a standard curve of total fluorescence intensity versus different concentrations of nanoparticle solution obtained via serial dilution of stock nanoparticle solution. The stock concentrations of 30 nm, 50 nm and 100 nm nanoparticle solutions are 1.6 × 10^15^/ml, 3.6 × 10^14^/ml, and 4.5 × 10^13^/ml, respectively. For example, 5 × 10^4^ 30 nm particles/cell, 2 × 10^4^ 50 nm particles/cell and 5 × 10^3^ 100 nm particles/cell were added to macrophage cultures, respectively. After incubating at 37 °C for 8, 16, or 24 hours, the macrophages were washed with PBS 2 times and imaged under an epifluorescence microscope (Leica) using a 60X water emersion objective equipped with a 580 ± 10 nm emission filter (PerkinElmer, MA). Phagocytosis index is determined by the median total fluorescence intensity measured per macrophage. Quantification of phagocytosis by flow cytometry was performed after the macrophages were detached from the culture dish and analyzed by the FACS Aria II flow cytometer (BD) for median fluorescence intensity of the cell population in the PE channel. All image analyses were done using the NIH ImageJ software.

### Confocal Microscopy

For confocal imaging, macrophages were plated in coverslip-bottom culture chambers (LabTEK) to confluence and fixed with 4% w/v formaldehyde for 30 min at room temperature. The cells were then wash with PBS for three times and incubated with primary antibodies of interest diluted in a blocking buffer according to manufacturer’s suggestion. Primary antibody incubation lasted for 3 hours and washed 3 times with PBST. At this time, the secondary antibodies conjugated with fluorescent probes were added and allowed to incubate for 3 hours. In the case for primary antibodies conjugated to a fluorophore, DAPI was added for nuclear stain. Cells were then washed with PBST 3 times and mounted with antifade mounting medium. Confocal microscopy was then performed with the Zeiss LSM 710 laser-scanning microscope using a 40x oil emersion objective, NA 1.2 (Carl Zeiss, Germany).

### Statistics

All data analyses were performed using the GraphPad Prism Software (La Jolla CA). For continuous variables, unpaired two-tailed t-test and one-way ANOVA were used to compare the means of 2 or more variables, respectively, with a p-value of 0.05 deemed as statistically significant. All measurements were performed at least 3 times.

## Additional Information

**How to cite this article**: Qie, Y. *et al.* Surface modification of nanoparticles enables selective evasion of phagocytic clearance by distinct macrophage phenotypes. *Sci. Rep.* **6**, 26269; doi: 10.1038/srep26269 (2016).

## Supplementary Material

Supplementary Information
