# Corrigendum: Surface modification of nanoparticles enables selective evasion of phagocytic clearance by distinct macrophage phenotypes

**DOI:** 10.1038/srep30663

**Published:** 2016-08-01

**Authors:** Yaqing Qie, Hengfeng Yuan, Christina A. von Roemeling, Yuanxin Chen, Xiujie Liu, Kevin D. Shih, Joshua A. Knight, Han W. Tun, Robert E. Wharen, Wen Jiang, Betty Y. S. Kim

Scientific Reports 6: Article number: 2626910.1038/srep26269; published online: 05 19 2016; updated: 08 01 2016

In this Article, Wen Jiang is incorrectly affiliated with:

Department of Hematology/Oncology, Mayo Clinic College of Medicine, 4500 San Pablo Road, Jacksonville FL, 32224, USA.

The correct affiliation is listed below:

Department of Radiation Oncology, UT-MD Anderson Cancer Center, 1515 Holcombe Blvd Houston TX, 77030, USA.

