# Supplementary material for: Surface modification of nanoparticles enables selective evasion of phagocytic clearance by distinct macrophage phenotypes
Source: Sci Rep. 2016 May 19;6:26269. doi: 10.1038/srep26269 (PMC4872535; doi:10.1038/srep26269)
Supplement: Supplementary Information [file srep26269-s1.pdf]

Surface modification of nanoparticles enables selective evasion of phagocytic clearance by distinct macrophage phenotypes

Qie Yaqing, Yuan Hengfeng, Von Roemeling A. Christina, Chen Yuanxin, Liu Xiujie, Shih Kevin, Knight A. Joshua, Tun W. Han, Wharen E. Robert, Jiang Wen, Kim Y.S. Betty

a

	Hydrodynamic diameter (nm)	SD	Charge (eV)	SD
30nm NP	37.8	4.2	-27.6	1.1
30nm NP-PEG	43.8	7.3	-24.3	1.5
30nm NP-CD47	46.9	8.1	-25.2	1.7
50nm NP	48.5	5.7	-28.9	1.4
50nm NP-PEG	55.6	6.9	-23.1	1.7
50nm NP-CD47	58.9	7.9	-29.2	2.1
100nm NP	99.2	8.1	-29.6	1.9
100nm NP-PEG	110.1	9.4	-26.1	1.8
100nm NP-CD47	114.7	10.6	-28.7	1.5

b

	Hydrodynamic diameter (nm)	SD	Charge (eV)	SD
30nm NP	39.6	4.5	-17.3	1.4
30nm NP-PEG	45.8	9.3	-17.4	1.6
30nm NP-CD47	45.4	10.1	-18.5	1.3
50nm NP	51.5	6.3	-17.6	1.2
50nm NP-PEG	57.3	7.6	-17.2	1.1
50nm NP-CD47	59.2	8.5	-18.4	1.6
100nm NP	96.2	10.2	-18.2	1.8
100nm NP-PEG	112.4	13.2	-17.4	1.4
100nm NP-CD47	117.3	14.7	-17.9	1.6

Supplementary Table 1: Size and charge characterization of unconjugated, 10K MW PEG conjugated, and CD47 conjugated nanoparticles in PBS a) and culture media b).

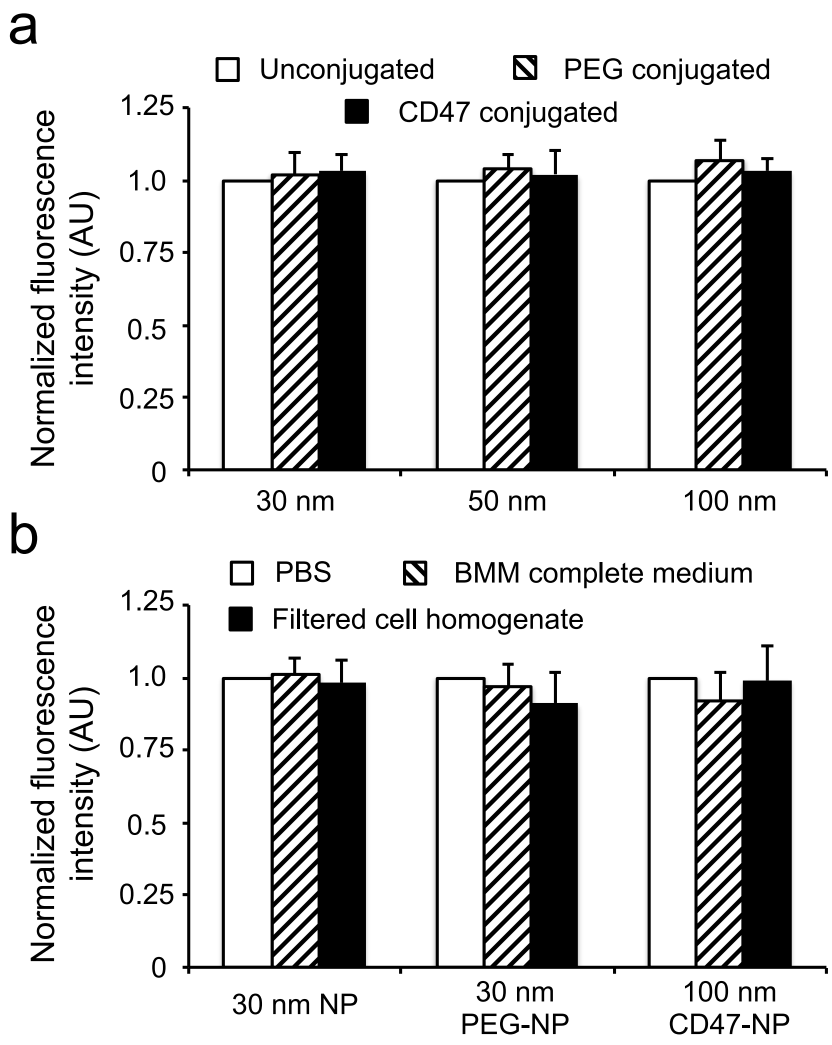

Supplementary Figure 1: Nanoparticles maintained stable fluorescence profile after conjugation with PEG or CD47 a) or post treatment with different incubation mediums for 8 hours b). Total fluorescence intensity were measured at standard dilution of the stock solution and normalized to unconjugated or PBS incubated nanoparticles. Data = mean, error bar = standard deviation, all measurements were repeated at least 3 times.

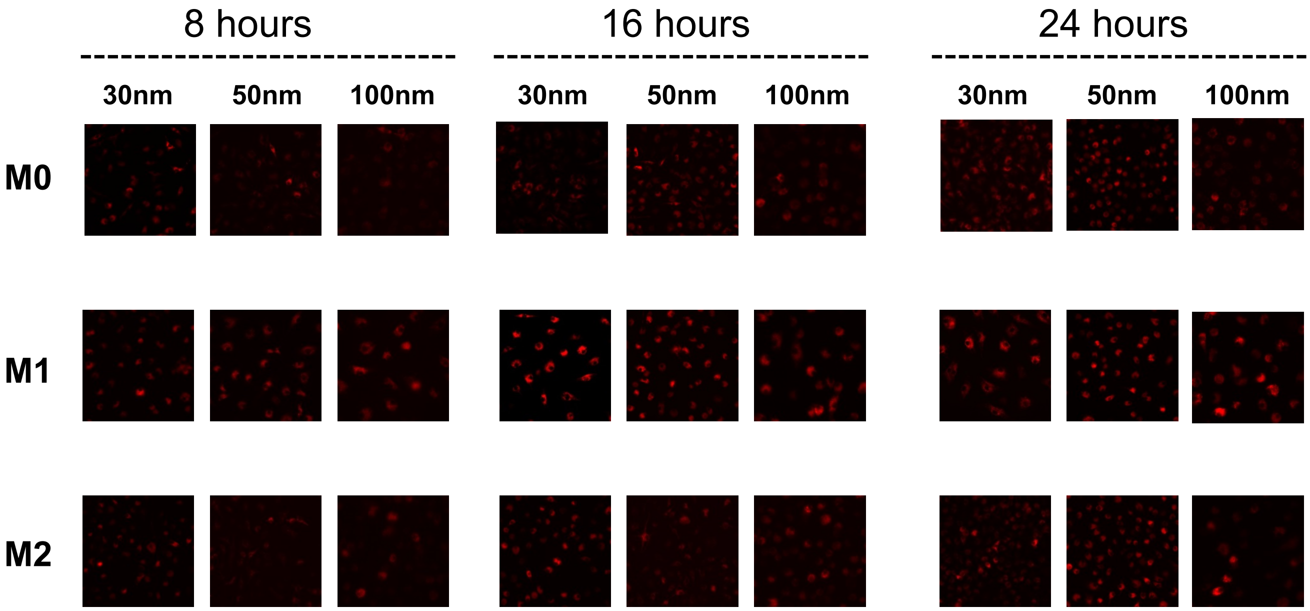

Supplementary Figure 2: Nanoparticle uptake by different macrophage populations with varying incubation times.

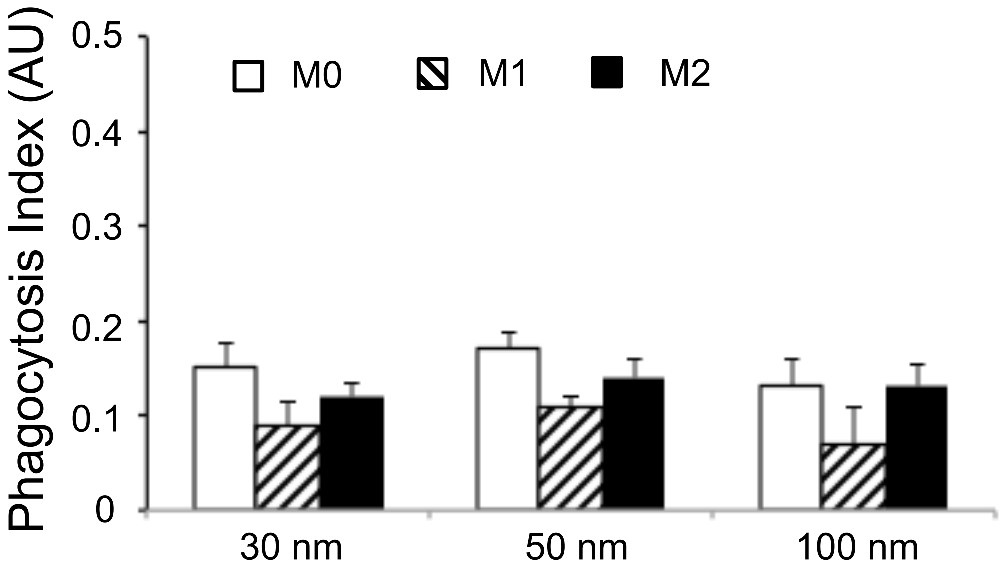

Supplementary Figure 3: Nanoparticle uptake by different macrophage populations when incubated at 4°C in the presence of 1% NaN_3. Phagocytosis index is determined by comparing to nanoparticle uptake by macrophages incubated at 37°C without NaN_3 treatment. Error bar = standard deviation, n=3.

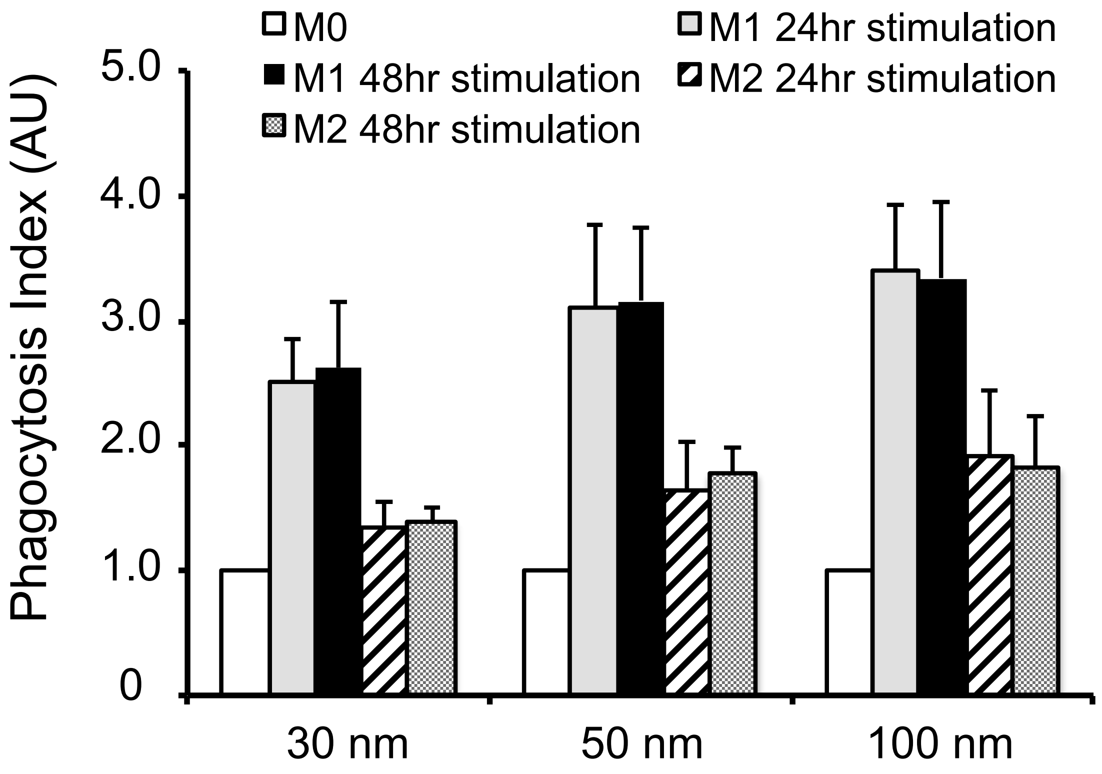

Supplementary Figure 4: Nanoparticle uptake by different macrophage populations with varying stimulation time. Stimulation with LPS or IL4 beyond 24 hours did not result in any significant difference in nanoparticle uptake by the macrophages. Data= mean, error bar = standard deviation, n=3.

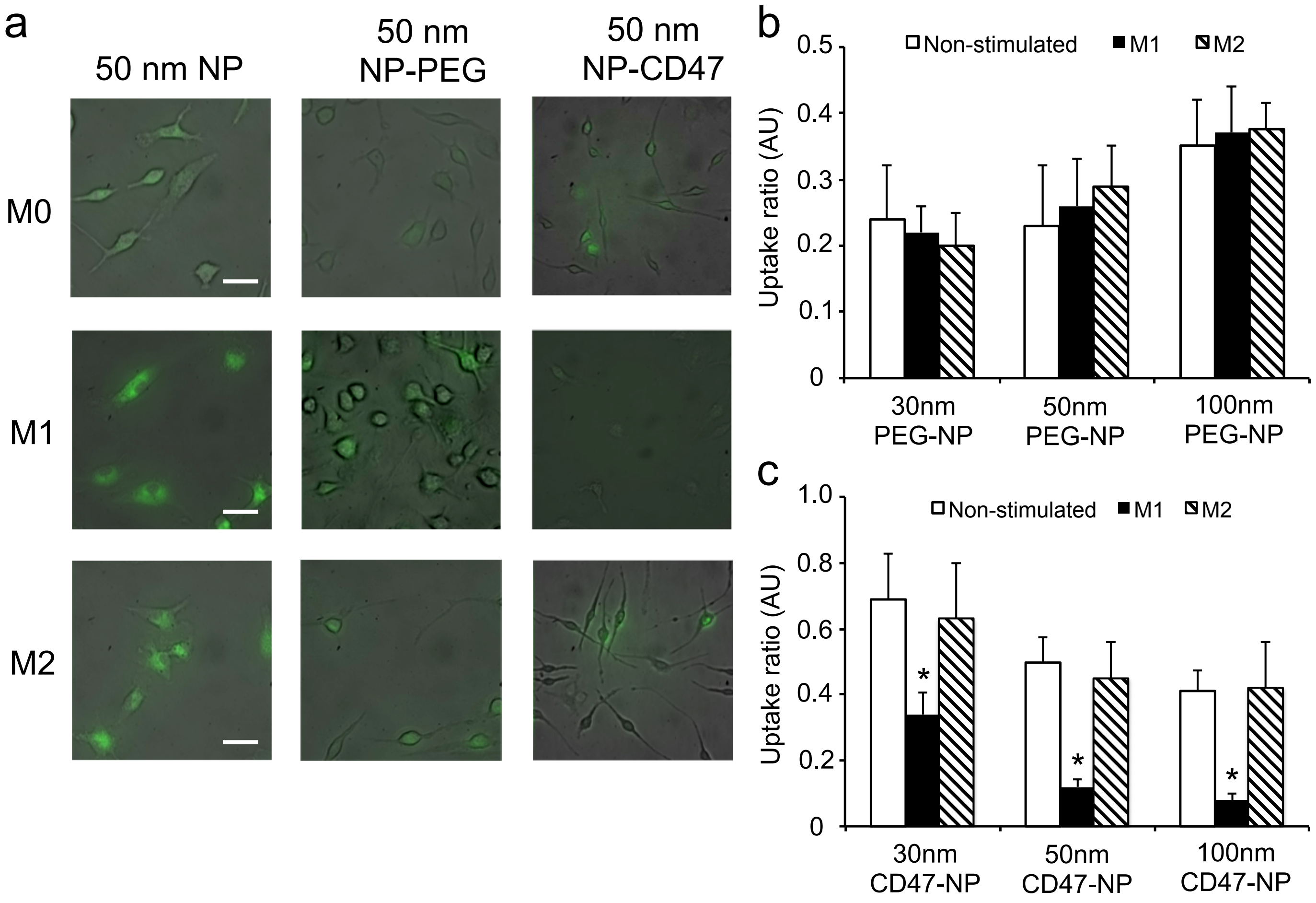

Supplementary Figure 5: Inhibition of macrophage nanoparticle uptake through surface modification. a) Fluorescent images of nanoparticle phagocytosis by different macrophage populations. Scale bar = 20μM. PEGlyation b) and surface coating with recombinant CD47 protein c) both decreased macrophage uptake as compared to non-surface modified nanoparticles based on flow cytometry analysis. CD47 conjugation, however, resulted in preferential decrease in uptake by M1 macrophages. Data =mean, error bar = standard deviation, n=3. * denotes $p<0.05$. Scale bar = 20μm.

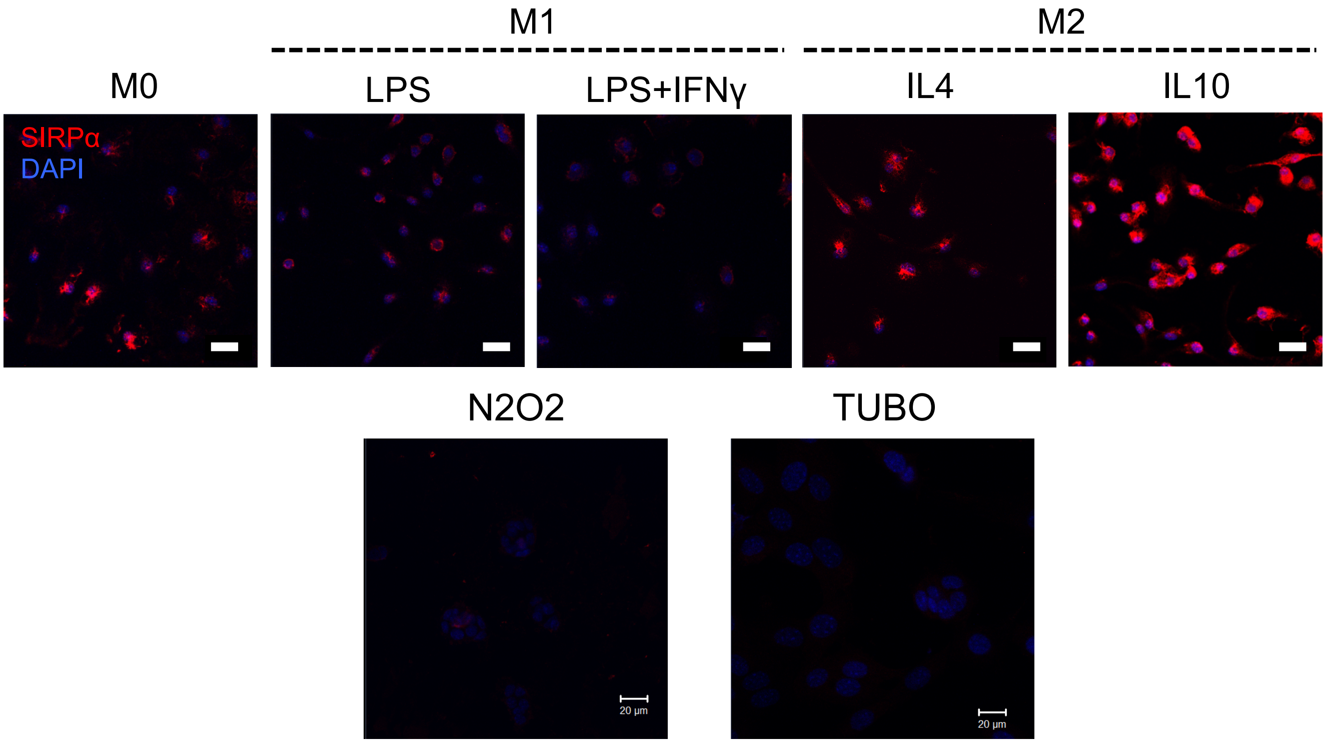

Supplementary Figure 6: Immunofluorescence staining of SIRPα expression in M0 and activated macrophages treated with different stimuli. Top) M1 macrophages exhibited significantly lower SIRPα expression compared to M0 and M2 macrophages. Bottom) Negative control for SIRPα expression using N2O2 and TUBO breast carcinoma cell lines showed minimal non-specific staining pattern for the antibody. Scale bar = 20µm.

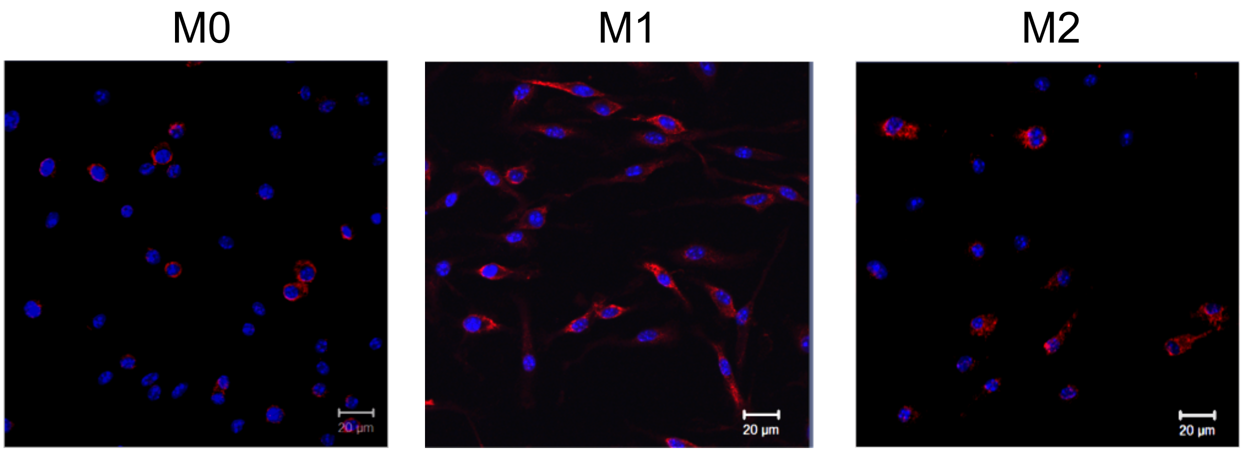

Supplementary Figure 7: Immunofluorescence staining of TSP-1 expression in M0 and activated M1 and M2 macrophages. Scale bar = 20μm.

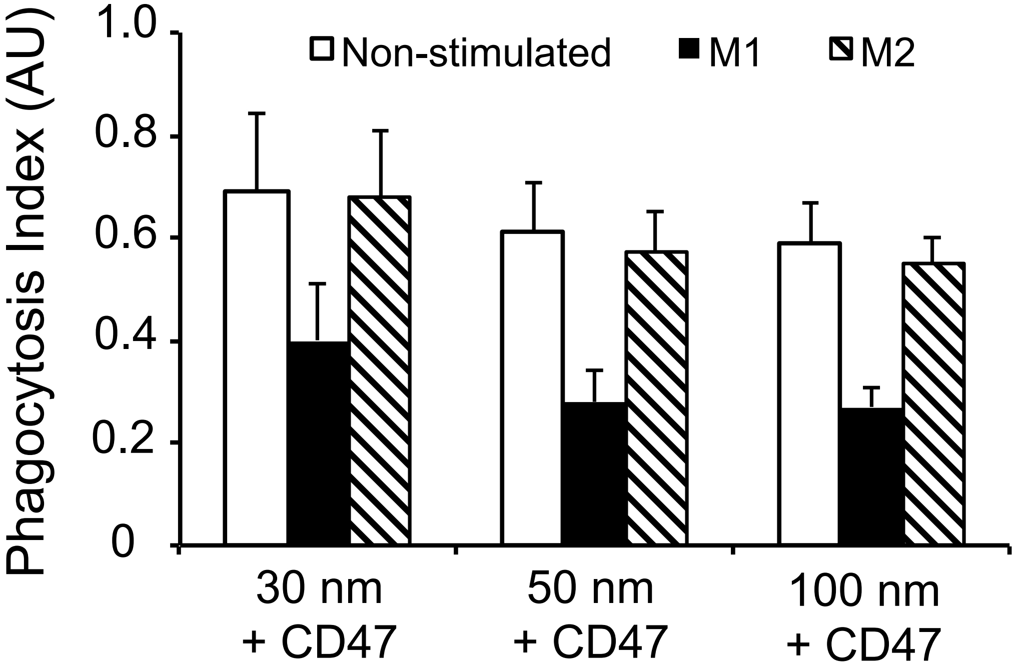

Supplementary Figure 8: Free CD47 reduced nanoparticle uptake by macrophages, but to a lesser degree compared to conjugated CD47-nanoparticles with the same CD47 dose and nanoparticle concentration. Data = mean, error bar = standard deviation, n =3.
